# Design of an Interpenetrating Polymeric Network Hydrogel Made of Calcium-Alginate from a Thermos-Sensitive Pluronic Template as a Thermal-Ionic Reversible Wound Dressing

**DOI:** 10.3390/polym12092138

**Published:** 2020-09-18

**Authors:** Hsiao-Ying Chou, Chang-Chih Weng, Juin-Yih Lai, Shuian-Yin Lin, Hsieh-Chih Tsai

**Affiliations:** 1 Graduate Institute of Applied Science and Technology, National Taiwan University of Science and Technology, Taipei 106, Taiwan; wherelove8@gmail.com (H.-Y.C.); wjg19wjg19@gmail.com (C.-C.W.); jylai@mail.ntust.edu.tw (J.-Y.L.); 2 Advanced Membrane Materials Center, National Taiwan University of Science and Technology, Taipei 106, Taiwan; 3 R&D Center for Membrane Technology, Chung Yuan Christian University, Chungli, Taoyuan 320, Taiwan; 4 Biomedical Technology and Device Research Center, Industrial Technology Research Institute, Hsinchu 310, Taiwan

**Keywords:** IPN hydrogel, PF127, alginate, VEGF

## Abstract

Polymer-based hydrogels demonstrate superior performance when used as wound dressing. An ideal dressing should possess an active healing function, absorb wound exudates, and provide a moist interface on the wound for rapid injury repair and the prevention of pain and injury during replacement of the dressing. Thus, the aim of this study was to develop a novel, reversible, smart, interpenetrating polymeric network (IPN) by utilizing the thermosensitive network of pluronic F127 (PF127) as a template to regulate the conformation of calcium-ion-crosslinked alginate. We found that the IPN hydrogels formed soft and elastic thermosensitive networks, retaining their form even after absorbing a large amount of wound exudate. The exterior of the hydrogels was made up of a rigid calcium alginate network that supported the entire hydrogel, promoting the stability of the vascular endothelial growth factor (VEGF) payload and controlling its release when the hydrogel was applied topically to wounds. Raman spectroscopy confirmed the layered structure of the hydrogel, which was found to easily disintegrate even after moderate rinsing of the wound with cold phosphate-buffered saline. Taken together, these results show that the IPN hydrogel developed in this study could be a promising delivery platform for growth factors to accelerate wound healing.

## 1. Introduction

Cutaneous wound healing is a complex physiological process comprising three main phases: inflammatory, proliferative, and remodeling [1,2,3]. The constant presence of injury or infection in the skin leads to a diminished healing ability and serious complications; hence, wound management is a critical clinical challenge. Injury to the skin—considered to be the body’s first line of defense—initiates an acute, protective cascade of events to avoid further damage to the underlying tissue [4,5]. In addition to the initial wound closure, wound healing is facilitated by a dressing that covers the injury and possesses an active healing function with coordinated recruitment of various angiogenic growth factors to the site of injury, thus accomplishing rapid repair and regeneration of the wound tissue. Angiogenesis is a crucial stage of wound repair where the formation of new blood vessels assists in the reconstruction of tissue by providing oxygen and nutrition [6,7]. The vascular endothelial growth factor (VEGF), one of the most potent pro-angiogenic cytokines, stimulates signaling in endothelial cells, chemotaxis, and angiogenesis [8,9]. Previous studies have demonstrated the up-regulation of VEGF within keratinocytes of skin wounds [10] and the resultant capillary formation [11,12,13]. Fibrin hydrogels covalently linked with VEGF have been shown to elicit a long-term angiogenic response in subcutaneous implants in mice [14]. For effective treatment of serious wounds, the combination of suitable materials of the wound dressing and growth factors has to be optimized to achieve synergistic potency. There are a variety of wound dressings, such as foam, fiber, hydrogel, and hydrocolloid, which have different performance characteristics, mechanisms, and functions [15]. Hydrogels have recently received much attention as appealing biomaterials that could serve as effective wound dressings by absorbing exudate fluid, creating a moist interface on the wound for better healing, and possessing a flexibility similar to that of human tissue [16,17].

Polymer-based hydrogels used for accelerating wound regeneration have replaced naturally derived materials owing to better performance and reproducibility [18]. Extensive research has been conducted on diverse types of pluronic hydrogels that could serve as a base owing to their thermosensitivity. To overcome their limitations of weak mechanical properties and swift erosion under physiological conditions, researchers have used chemical crosslinking or the formation of physical mixtures by blending in proteins, polysaccharides, and semi-interpenetrating polymeric networks (SIPNs) [19,20]. Thus, hydrogels consisting of a physical mixture of pluronic and hyaluronic acid (HA) [21,22], HA chemically grafted onto pluronic [23], a combination of alginate, pluronic, and HA [24], or chemically grafted or physically blended alginate and pluronic [25,26] could exhibit gelation behavior and serve as vehicles for long-term delivery of drugs or cells. Alginate gel formation can be induced by phase transition, free radical polymerization, and various crosslinking methods—ionic, cell, and covalent crosslinking [27,28,29,30]. Ionic crosslinking is the most common gelation method to form alginate hydrogels by creating egg-box structures via ionic bridges with divalent cations (Ca^2+^, Mg^2+^, Sr^2+^); utilizing lactones to reduce the pH value below the pKa of alginate monomers is another alginate gelation method [31,32]. Although the preparation of calcium alginate hydrogels is both simple and cost-effective, network heterogeneity inside a conventional calcium alginate hydrogel results in a wide distribution of mesh sizes [33]. The intermolecular crosslinking between calcium chloride and alginate enhances the mechanical strength of the gel by impeding its degradation in the body [34,35]. Another drawback of calcium alginate hydrogels is high porosity that leads to a low retention capacity for payload molecules [36,37]. 

To overcome the limitations of single-component hydrogels, hydrogels have been synthesized using nanocomposite, hybrid, and interpenetrating polymeric network (IPN) networks that endow the gels with superior and new functions. Polymeric network hydrogels have been found to possess greater mechanical strength and are able to absorb a large volume of wound exudate [16,30,38], while retaining their architecture, performance, and stability at the interface of the wound. They also minimize pain and further injury to the patient while changing the dressing. A template of Pluronic F127 (PF127) could control gel formation and alginate arrangement, resulting in thermo-reversibility and without compromising gel performance, and facilitating easy wound management and efficient recovery.

A polymeric blend or grafted pluronic have been shown to be helpful in improving stability and gelation behavior. However, few studies have focused on the reversibility of hydrogels and their applications. The objective of this study was to develop a novel, smart, reversible-response IPN hydrogel as a functional wound dressing with pro-angiogenic activity to accelerate wound healing. Briefly, from the pluronic series of polymers, nonionic PF127 was selected as the basis of the hydrogel system because of its simple phase behavior and low gelation concentration [20]. We used both alginate (because of its biocompatibility) and PF127 to develop an IPN (PF127/SA) hydrogel platform that would release its drug payload for wound treatment. Regarding the alginate and PF127 combined hydrogel, the inclusion of a thermos-sensitive network and the calcium ion crosslinked in our IPN network, distinguish it from the fabrication processes described in previous research. In the meanwhile, the VEGF, which is recognized as the best-characterized endothelial-specific growth factor, is encapsulated into the IPN network hydrogel. We further studied the thermo-sensitive gelation properties of PF127, as well as the morphology, stability, and sustained release of VEGF in the presence of crosslinked IPN. In addition, we examined whether the IPN network hydrogel could sol–gel phase transition and easily dispose of a network by exposing it to a cold ionic buffer in vitro and in vivo, thus benefiting wound treatment circumventing lacerations and further injury. 

## 2. Method

### 2.1. Materials

Pluronic F127 (PF127, Mn: ~12600 g/mol), sodium alginate (W201502, alginic acid sodium salt from brown algae and with an M/G ratio of 1.61 determined by NMR analysis [39]), and a mouse VEGF enzyme-linked immunosorbent assay (ELISA) kit for serum, plasma, and cell culture superatats (RAB0509) were purchased from Sigma-Aldrich (Saint Louis, MO, USA). Calcium sulfate was purchased from Fisher Scientific (Waltham, MA, USA). Sterilized phosphate-buffered saline (PBS), 3-(4,5-dimethylthiazol-2-yl)-2,5-diphenyl-tetrazolium bromide (MTT), Dulbecco’s modified Eagle’s medium (DMEM), fetal bovine serum (FBS), penicillin, sodium pyruvate, and trypsin-ethylenediaminetetraacetic acid (EDTA) (0.25%) were purchased from Gibco (Carlsbad, CA, USA). Cellulose dialysis membrane (molecular cutoff (MWCO) of 6−8 kilodalton (KDa)) was purchased from Orange Scientific (Braine-l'Alleud, Belgium). Deionized water was obtained using a Millipore water purification system.

### 2.2. Preparation of Polymeric Blended Hydrogels

The Pluronic incorporated sodium alginate (PF127/SA) hydrogels were prepared by forming an IPN network of calcium-ion-crosslinked sodium alginate and thermosensitive pluronic PF127. Briefly, a physical mixture of PF127 and SA was blended with various weights per volume concentration (% *w/v*) of PF127/SA of 15/0.25, 15/0.5, 15/1, and 15/1.5 by dissolving it in distilled water under constant stirring in a 4 °C water bath to ensure complete dissolution. The homogeneous solution was placed in a dialysis membrane (MWCO 6–8 KDa) and dialyzed for 24 h against a calcium sulfate slurry (5 gL^−1^) preheated to 37 °C. 

### 2.3. Sol–Gel Phase Transition Behavior

Sol–gel phase transitions of the aqueous triblock PF127-blended SA were investigated using (i) the tube-inversion method and (ii) Brownian motion, which was observed during the gelation process by dynamic light scattering (DLS). First, the gelation temperature was tuned with different hydrogel solution concentrations: solutions of PF127 of concentrations ranging from 12.5 to 27.5% *w/v* were mixed with predefined SA solutions containing 0 to 1.5% *w/v*. Thereafter, the sol–gel transition behavior was recorded by using the following procedure [25,40,41]. Five milliliters of the homogeneous solution in a vial test tube were placed at 0 °C for 24 h and then placed in an interval-controlled temperature water bath for 10 min with a temperature interval of 5 °C, at a heating rate of 1 °C/min. This was followed by continuous tube inversions under observation, and the gelation temperature was recorded when no solution flowed downwards within 60 sec of inversion. Furthermore, we used DLS to investigate the temperature-dependent sol–gel phase transition of different weights per volume concentration (% *w/v*) of PF127/SA of 15/0.25, 15/0.5, 15/1.0, and 15/1.5 as a function of temperature. The mixtures were gradually heated from 20 °C to 50 °C at an interval of 5 °C, and the correlation function was monitored during the gelation of the samples.

### 2.4. Physical Characteristics

The characteristic analysis of the PF-127/SA hydrogel was prepared through the combination of thermosensitive and calcium-ion crosslinking. Field-emission scanning electron microscopy (FESEM, JSM 6500F, JEOL, Tokyo, Japan) and Raman spectroscopy (JASCO NRS5100, Easton, MD, USA) with a laser light wavelength of 532 nm were used to observe the nanostructural morphology of PF-127/SA hydrogels. Briefly, the hydrogel was frozen and lyophilized. Then, a specimen was fractured in liquid nitrogen and sputtered with platinum before obtaining an image of the hydrogel. The mixture solutions of PF127 and SA were dialyzed against a CaSO_4_ slurry at 37 °C, and the PF127/SA IPN hydrogel was obtained for Raman measurement. The viscosity of these hydrogels was measured at a shear rate of 0.1 (1/s) using a modular compact rheometer (MCR102) with increasing temperatures (20–70 °C, at a heating rate of 5 °C min^−1^). Evaluation of hydrogel reversibility was done by placing the hydrogel in 0.1 M PBS at 4 °C and observing the phase transition using photographs.

### 2.5. In Vitro Cytotoxicity Test (MTT Assay)

The cytotoxicity of PF127/SA hydrogels, after gelation, was evaluated by using the MTT assay using human keratinocytes (HaCaT cells) grown in DMEM supplemented with 10% FBS at 37 °C in a humidified atmosphere of 5% CO_2_. HaCaT cells were seeded in 96-well plates at 1 × 10^4^ cells/well, and after overnight incubation, the cells were washed and incubated with different extracts for another 24 h. These extracts were obtained as follows: the IPN network PF-127/SA hydrogel was sterilized with Gamma radiation for 30 min and immersed in a prepared medium at 37 °C for 24 h [42]. After incubating the cells with extracts, the cells were incubated with MTT (0.5 mg/mL) for 4 h. The supernatant medium was subsequently replaced with dimethyl sulfoxide (DMSO) to dissolve the formazan crystals formed in viable cells. The absorbance (570 nm) was measured using an ELISA plate reader (ELISA Reader, Thermo Multiskan FC Microplate Photometer, Waltham, MA, USA), and cell viability was calculated using the following Equation (1):

(1) $$\mathrm{Cell}\;\mathrm{Viability}\;(\%)\;=\;\frac{\mathrm{absorbance}\;\mathrm{of}\;\mathrm{treated}\;\mathrm{cells}}{\mathrm{absorbance}\;\mathrm{of}\;\mathrm{control}\;\mathrm{cells}}\times 100\%$$

### 2.6. In Vitro VEGF Release Study

The VEGF (1 μg/mL) was dropped into a vehicle solution (PF127/SA 15/0.25), while the homogeneous solution was dialyzed (MWCO 6–8 KDa) against 37 °C of calcium sulfate slurry over 24 h to form the hydrogel. The VEGF-loaded IPN hydrogel (1 × 1 cm) was immersed in 10 mL of pH 7.4 PBS at 37 °C and shaken at 100 rpm. Then, 2 mL of the medium was collected at predetermined intervals. This system was maintained at a constant volume with a fresh volume of PBS. A mouse VEGF ELISA kit was used to evaluate the VEGF release profile against the PBS at 37 °C according to the manufacturer’s instructions, and the absorbance (450 nm) was measured using an ELISA plate reader (ELISA Reader, Thermo Multiskan FC Microplate Photometer, Waltham, MA, USA). The experiment was performed in triplicate, and the cumulative release was calculated by using the Equation (2):

(2) $$\mathrm{Cumulative}\;\mathrm{release}\;(\%)=\frac{\mathrm{Concentrationof}\;\mathrm{drug}\;\mathrm{release}}{\mathrm{Concentrationof}\;\mathrm{drug}\;\mathrm{payload}}\times 100\%$$

### 2.7. In Vivo Animal Model

The wound healing efficiency of the PF127/SA-VEGF hydrogel was assessed in 6–8-week old female BALB/c mice purchased from BioLASCO (Taiwan Co., Ltd., Taipei, Taiwan). All animal care and handling procedures were carried out in compliance with the approved guidelines of National Applied Research Laboratories (NARLabs), National Laboratory Animal Center. Briefly, 6 mm-diameter skin wounds were created on each mouse by using a biopsy punch under aseptic conditions. The wound surface was covered with different types of hydrogel dressings (PF127/SA-VEGF, PF127/SA, kaltostat, and control). At intervals of 2 to 3 days, cold PBS was used to wash the dressing off the wound, and a new dressing was placed on the wound. Wound assessment was performed using photographs, pathological analyses with hematoxylin and eosin (H&E) staining, and wound closure efficiency according to the following formula [1,43] (3):

(3) $$\mathrm{Cure}\;\mathrm{area}\;(\%)\;=\;1-\;\frac{\mathrm{wound}\;\mathrm{area}\;\mathrm{on}\;\mathrm{day}\;\mathrm{one}-\mathrm{wound}\;\mathrm{area}\;\mathrm{on}\;\mathrm{day}\;\mathrm{of}\;\mathrm{assessment}}{\mathrm{wound}\;\mathrm{area}\;\mathrm{of}\;\mathrm{day}\;\mathrm{one}}\times 100\%$$

## 3. Results and Discussion

The two types of networks, including crosslinked calcium alginate and the thermosensitive network of PF127, present in the PF127/SA IPN hydrogel, were prepared by dialyzing a polymeric blended solution at 37 °C against a calcium sulfate slurry. This method of fabrication is different from that used in a previous study in which the calcium alginate was first crosslinked and subsequently allowed to interact with other polymers to create the second structure. Herein, the main aim of our study was to utilize the template of PF127 to arrange the gelling conformation in SA while further examining some of the features and functions of the hydrogels based on the amount of SA incorporation, gelation condition, morphology, and effective wound healing.

**Figure 1:**
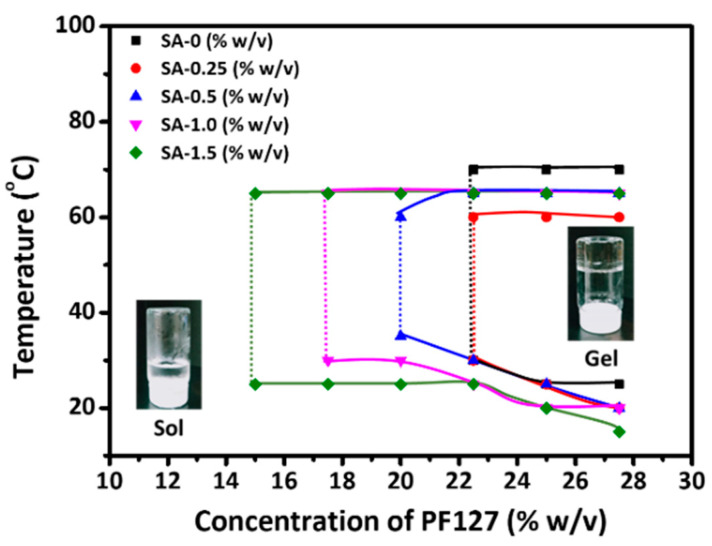
Sol–gel transition diagram of a reversible thermosensitive hybrid hydrogel, prepared by blending SA into PF127. The hybrid hydrogel consisting of different concentrations of PF127 (12.5–27.5% *w/v*) and amounts of SA (0–1.5% *w/v*) was subsequently assessed by using the tube-inversion method at increasing temperature (5–70 °C) (three measurements for each point). SA, sodium alginate

PF127 is an amphiphilic, triblock copolymer utilized as a potential thermosensitive hydrogel at a specific temperature [44]. The characteristic lower critical solution temperature (LCST) of the hydrogel can be tailored by controlling the SA concentration and amount of PF127 during fabrication of the hydrogel. Figure 1 shows the sol–gel transition behavior of PF127/SA polymeric, blended hydrogels containing various amounts of SA over a temperature range. The gelation point shifted towards lower temperatures with a gradual increase in the proportion of SA from 0 to 1.5% *w/v*, while the gelation temperature extended over a broader range, and vice versa. Moreover, increasing the amount of SA could effectively induce hydrogel formation and reduce the PF127 concentration required for hydrogel formation. This could be attributed to the strong entanglement of PF127 and SA wherein the high content of SA could bind to the polyoxyethylene chains present in PF127. This interaction induced the dehydration of PF127 and further increased the hydrogen bonding interaction, thereby resulting in hydrogel formation [21,45]. These results show that the LCST of the PF127/SA hydrogel depended on the number of SA molecules, and that the thermosensitive property of PF127 was not affected by the addition of polysaccharide SA.

**Figure 2:**
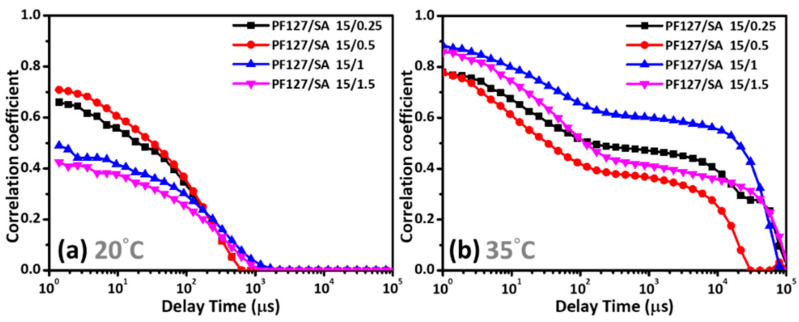
SA concentration effects on the correlation coefficients of thermosensitive PF127/SA hydrogels at (**a**) 20 °C and (**b**) 35 °C. The correlation coefficient as a function of time was obtained by dynamic light scattering measurements for various temperatures.

Hydrogel is a multi-component system with viscoelastic properties [46]. In the presence of SA, the phase transition behavior of 15% *w/v* PF127 solution was recorded as a function of temperature by using DLS (Figure 2). Despite the presence of SA, temperature-induced PF127/SA hydrogel formation still continued. In all systems, the correlation coefficient exhibited fast exponential decay and a single-mode characteristic at a low temperature of 20 °C (Figure 2a, sol state), while slow multi-mode relaxation appeared at a corresponding gel temperature of 35 °C (Figure 2b, inter-micellar aggregates and arrange to gel state). This result is in agreement with previous findings that PF127 is soluble in aqueous solution at low temperatures and forms a gel with increasing temperature [20,47]. 

In fact, the correlation coefficient indicated a pronounced fast dynamic decay below the critical gelation temperature (CGT) in solution, possessed kinetic energy and showed continuous Brownian motion. Above the CGT, the progressive dehydration of polypropylene oxide (PPO) chains, breaking of hydrogen bonds, and strong intermolecular entanglement result in a gel state via micelle aggregation and arrangement [44,48]. Therefore, Brownian motion slows down and the enhanced dynamical constraints (correlation functions close to 1), resulting from additional intermicellar interplay, form a gel. Figure 2a shows that varying the amount of SA hardly changed the sol state at low temperatures. However, increasing amounts of SA in the PF127/SA mixture reduced the CGT values to favor micellar aggregation (Figure S1), which is consistent with the sol–gel transition profiles. 

**Scheme 1:**
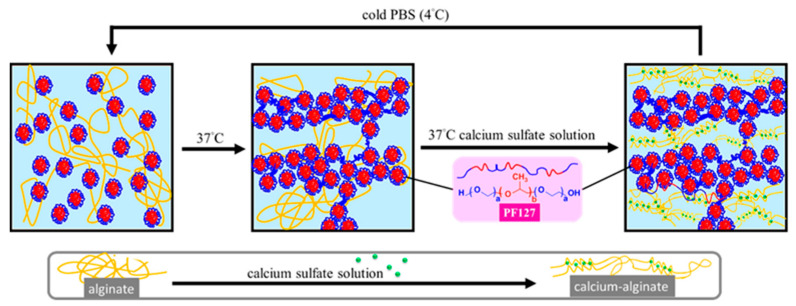
A novel reversible interpenetrating polymeric network hydrogel is made up of a thermosensitive network and calcium-ion-crosslinked alginate.

The thermosensitivity of the polymeric, blended hydrogel was affected by the amount of the polysaccharide, SA. The use of calcium sulfate slurry at 37 °C in this study, instead of the commonly used calcium chloride, provided retarded gelation, a uniform gel structure, and high strength, which was different from the fabrication process and results of previous studies. As illustrated in Scheme 1, the calcium ions hold the alginate molecules together to form a specific egg-box network for alginate formation after the arrangement of PF127 molecules in a closely packed cubic structure. As a result, the PF127/SA hydrogels are made up of two types of crosslinked networks formed via a thermosensitive process and the addition of calcium ions. We analyzed the gelation features and structures of these dual-network hydrogels.

**Figure 3:**
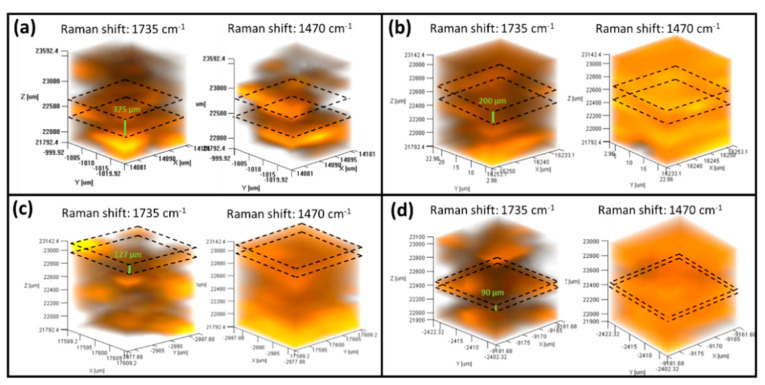
3D Raman mapping images of the combined ion- and thermally crosslinked dual-polymeric hydrogels. All of these hydrogels comprise weights per volume concentration (% *w/v*) of (**a**) 15/1.5, (**b**) 15/1.0, (**c**) 15/0.5, and (**d**) 15/0.25 (abundant alginate between boundaries).

**Figure 4:**
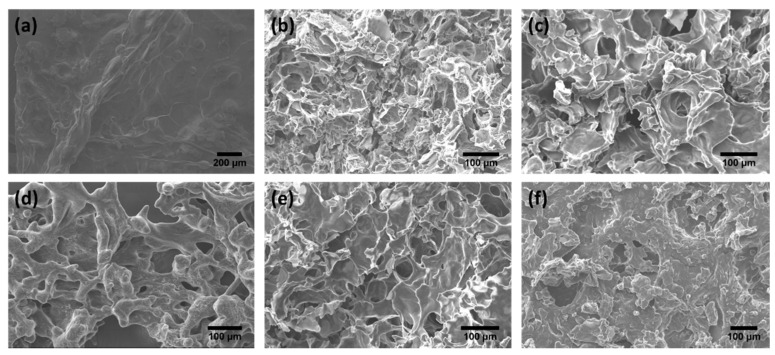
Cross-sectional scanning of different hydrogels from SEM images after the freeze-drying process, including single networks of (**a**) 1.5% *w/v* SA with calcium ion crosslinked and (**b**) 15% *w/v* PF127; and IPN network hydrogels composed of PF127/SA *w/v* of (**c**) 15/0.25, (**d**) 15/0.5, (**e**) 15/1.0, and (**f**) 15/1.5.

The spatial organization of the hydrogels was analyzed using Raman spectroscopy because of this method’s low sensitivity to the hydrated samples and its nondestructive nature [49]. First, the characteristic peak at 1470 cm^−1^ was assigned to the Poly(ethylene oxide) (PEO) chain of PF127 and the peak at 1735 cm^−1^ corresponding to SA was identified in reflected 3D Raman mapping from 2D Raman spectra (Figure S2). PF127 formed the highly dense structure of the hydrogel at 37 °C via the formation of self-assembled micelles, following which the layered structure of SA was inserted between the closely packed cubic arrangement of PF127 (Figure 3). First, the networks within the hydrogel formed the frame via thermal crosslinking, after which calcium alginate created a second structure along the PF127 gel via a slow ion-exchange process. The large amounts of SA emerged at the boundary, while the distance between layers was gradually reduced from 375 to 90 μm (Figure 3). Indeed, the hydrogel exhibited an apparently layered structure with respect to the higher proportion of SA present (Figure 3a). The exterior of the hydrogel consisted of a calcium alginate network that could be regarded as a framework for supporting the overall hydrogel structure. The cross-sectional morphology of the hydrogel was examined by SEM (Figure 4). The PF127 was found to be assembled into a transient polymeric network in all hydrogels, which is in agreement with the findings of a previous study [25,26]. Some of the irregularly interconnected texture and large pores present in the IPN network matrix confirmed inter-chain interaction. Such a structure is advantageous for wound treatment as this system could remain permeable to oxygen and absorb wound exudate.

**Figure 5:**
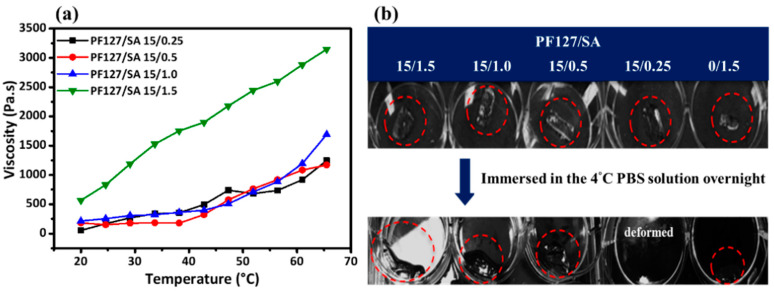
Effects of the IPN on mechanical and reversible properties determined using a rheometer: (**a**) at different temperatures under the same shear rate (0.1 s^–1^); (**b**) photographs of PF127/SA hydrogel reversible test at 4 °C (below lower critical solution temperature -LCST) (single crosslinked calcium alginate was used as a control group).

The reversible characteristics of this dual-network hydrogel and the combination of calcium alginate and thermally crosslinked PF127 are critical factors for wound management. The sol–gel transition behavior is accompanied by a change in viscosity, which is the tendency of the fluid to resist flow [50,51]. Hence, the reversible property of the IPN hydrogel, which is strongly dependent on the applied temperature, is described by viscoelasticity. Among all the dual-network hydrogels, uniform IPN structure, composed of ionic crosslinked and physical networks, and the highest strength were observed for the hydrogels fabricated with PF127/SA 15/1.5 (Figure 5a). Large amounts of SA strengthened the polymeric interactions and increased the viscosity of the system, as reported previously [45]. When the temperature decreased from 35 to 20 °C, an abrupt decrease in viscosity to close to 0 (low viscosity means easy flow) indicated a sol–gel phase transition, especially in the PF127/SA 15/0.25 hydrogel, resulting in the lowest viscosity among all the fabricated hydrogels and confirming the reversible property of the IPN hydrogel triggered by temperature (Figure 5a). This result is ascribed to the presence of a solution state below the critical gelation temperature (CGT) together with a weak network with SA and a low degree of ionic crosslinking, resulting in easier deformation under continuous shear stress (the progressive breakdown of networks into smaller clusters leads to little friction in the molecular makeup when it is in motion [52,53]). Finally, low-viscosity liquids rarely show resistance to flow and thus liquid momentum homogeneously disperses integrated systems, indicating that a low energy is required for deformation. When the temperature was decreased to 4 °C, the deformation behavior of the IPN in the PF127/SA 15/0.25 hydrogel was remarkable (Figure 5b). By contrast, the single ionic crosslinked alginate hydrogel remained intact and retained an irregular shape when the temperature was decreased to 4 °C. This could be attributed to the high degree of relatively rigid ionic crosslinking, leading to poor dissociation of the polymeric network. In terms of the phase transition from gel to solution, the dual-network hydrogels could be easily rinsed away by washing with cold PBS (a temperature below LCST (4 °C)). Based on its physical features, optimal reversibility was observed for the PF127/SA 15/0.25 hydrogel; hence, it was selected for use in the in vivo experiments.

**Figure 6:**
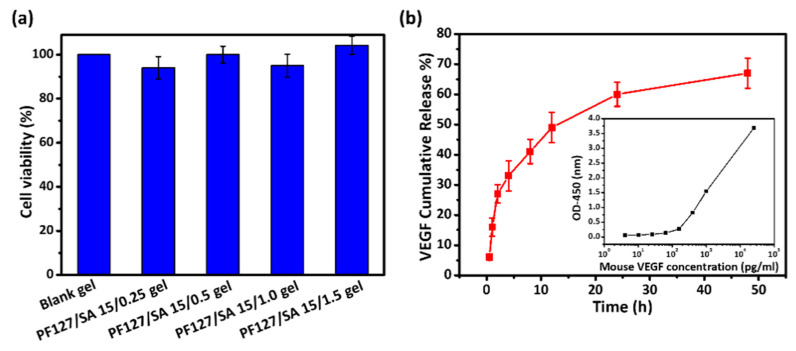
(**a**) In vitro cytotoxicity of PF127/SA IPN hydrogels. MTT assay was used to determine the concentration-dependent cell viability of HaCaT cells after 24 h of treatment with extracts after gelation (hydrogel state, with calcium) while the blank gel was only treated with DMEM (as a control). (**b**) In vitro VEGF release profile from PF127/SA IPN hydrogels over 48 h at 37 °C. The inset in (**b**) of a standard curve was measured following the manufacturer’s recommendation. (MTT, (3-(4,5-Dimethylthiazol-2-yl)-2,5-diphenyltetrazolium bromide); DMEM, Dulbecco's Modified Eagle Medium; VEGF, Vascular endothelial growth factor)

Both SA and PF127 are United States Food and Drug Administration-approved biocompatible polymeric materials and are widely used in several clinical applications [24,54,55]. However, the cytotoxicity of hydrogels is a critical consideration before performing in vivo experiments because of the potential influence of the crosslinking calcium ions on cell proliferation and survival. The considerably high cell viability observed after treatment of HaCaT cells with PF127 blended with SA (Figure 6a) demonstrated the biocompatibility of the polymeric materials after gelation (such as IPN hydrogels), which fulfills the criteria for the biological evaluation of medical devices mentioned in the ISO 10993-5 [56] standard for these tests. 

Because of the surfactant properties of PF-127, both hydrophilic and hydrophobic drugs could be encapsulated in their hydrogels [57]. Potential drawbacks of PF-127 hydrogels include rapid erosion and weak mechanical strength after absorption of the exudate tissue fluid on the wound, which results in instability and burst drug release [21,22,24]. Therefore, the PF-127 hydrogel is not suitable for drug delivery. Hence, the physically blending polysaccharide SA into a PF127 solution could improve the mechanical strength of the hydrogel, into which VEGF could be encapsulated for controlled release. This change in the physical and release behavior of the hydrogel could be attributed to the conformation and the reversible electrostatic binding interactions with SA in the hydrogel [18,58]. Figure 6b shows the steady sustained VEGF release from PF127/SA IPN hydrogels over time (the cumulative release was ~67%) via ion exchange and a concentration gradient. Thus, the barrier of calcium alginate had no significant effect on VEGF release from the hydrogels, whereas it slowed down the release rate (by inhibiting the burst release phenomenon). The system combining a PF127 physical network and calcium-crosslinked alginate shows great promise as a potential drug delivery platform.

**Figure 7:**
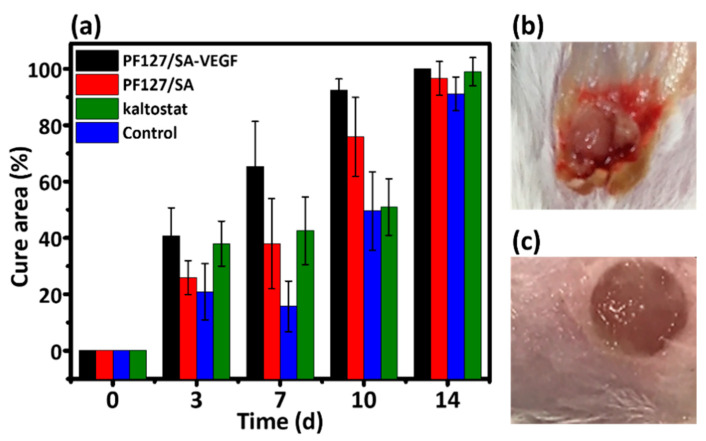
(**a**) Evaluation of cure efficiency on wounds after treatment with various types of wound dressings over a 14 d period. Photographs of the wound healing process treated with (**b**) control group versus (**c**) PF127/SA IPN hydrogel.

Based on the release kinetics of VEGF—up to 67% released over 2 d—the hydrogel was to be replaced every 2–3 d in our in vivo experiments. Generally, wounds take approximately two weeks to heal. Hence, we treated the wounds with the different hydrogels as wound dressing and recorded the results of wound repair for two weeks. We observed faster healing kinetics with hydrogels containing alginate than with those without alginate in the control group (Figure 7a), as the alginate hydrogels triggered cytokine production and macrophage activity in the wound site [59]. In addition, calcium alginate can promote cellular activity and proliferation by releasing calcium onto the wound [60,61,62]. Of note are the severe laceration and bleeding caused by the adhesion between the wound and the dressing in the control group (Figure 7b), which resulted in an expansion of the wound in the control group at 7 d. By contrast, Figure 7c shows the intact wound because of the reversible behavior of the PF127/SA hydrogel system that allowed easy removal by rinsing the wound with cold PBS. No significant difference was observed in the presence of alginate hydrogels (Figure 7a). We examined the healing efficiency of the different types of hydrogels (PF127/SA and kaltostat) over a period of two weeks. The most rapid cure efficiency was observed for wounds treated with IPN hydrogel with the VEGF payload: 92% healing was observed on day 10. Taken together, these results indicate that the delivery of VEGF onto the wound by the PF127/SA IPN hydrogel system stimulated vascular endothelial cell proliferation and prompted granulation tissue formation for wound recovery, which is consistent with previous findings [63,64]. We also examined the wound tissue using histological analysis.

**Figure 8:**
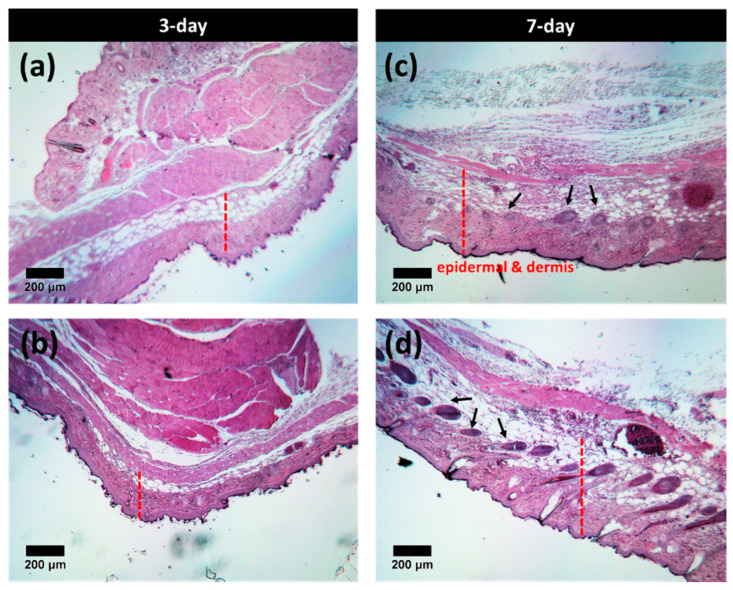
Photomicrographs of histological comparison between PF127/SA IPN hydrogels (**a**,**c**) without VEGF and (**b**,**d**) with VEGF for mouse wound healing. Each sample was taken after 3 d and 7 d intervals and stained with H&E (new tissue generation marked with an arrow; the red dashed line indicates thickness of the tissue). H&E, hematoxylin and eosin stain

Figure 8 shows the histological structure of the wound tissue treated with IPN hydrogels with and without incorporated VEGF. No significant discrepancy was noted in the appearance of the wound tissue for treatment of hydrogel without (Figure 8a) and with VEGF (Figure 8b) on day 3. However, on day 7, re-growth of normal hair follicles and generation of sebaceous glands in the dermis were observed along with the absence of an inflammatory cell infiltrate in the wound tissue of mice treated with hydrogels with and without VEGF (Figure 8c,d). Notably, higher levels of granulation tissue formation were observed in the mice treated with hydrogels with a VEGF payload than in those treated with hydrogels without VEGF. The presence of fibroblasts, mature hair follicles, and collagen in the dermis layer confirmed complete wound healing [65]. Thus, VEGF endowed promoting tissue regeneration and in vivo wound healing activity in the system. To sum up, these observations demonstrate chemotactic activity for inflammatory cells and stimulation of tissue re-growth by alginate-triggered cytokines and growth factors, indicating favorable synergistic effects of PF127/SA IPN hydrogel-delivered VEGF for wound healing.

## 4. Conclusions

We developed a novel, reversible IPN hydrogel, composed of physical, thermosensitive networks and ionic crosslinking, and demonstrated its safety and biocompatibility. Despite the presence of sodium alginate within the PF127 hydrogels, the thermosensitive PF127 network still promoted gelation and transformed the LCST range. The introduction of calcium ions into the hydrogel resulted in the formation of a second network via ion exchange, thereby creating an elastic, layered structure in the interior and a rigid network on the exterior of the hydrogel. Additionally, we observed that with a reduction in the amount of sodium alginate in the hydrogel, even moderate rinsing with cold PBS allowed easy removal of the hydrogel by its conversion from the gel state to the solution state. The construction of the PF127 template hydrogel favored the encapsulation and sustained delivery of active VEGF onto the wound. Our results confirm that the IPN hydrogel provided a moist interface on the wound, the VEGF release promoted rapid wound repair, and the behavior of the gel facilitated wound debridement via a simple rinsing process. In conclusion, these IPN PF127/SA hydrogels show great promise as a delivery platform of various payload drugs for wound therapy, and their properties will help avoid further laceration and pain during wound management.

## Supplementary Materials

The following are available online at https://www.mdpi.com/2073-4360/12/9/2138/s1, Figure S1: The effect of the sol-gel transition behavior with different temperature in IPN hydrogel, Figure S2: 2D Raman spectrum of PF127/SA IPN hydrogel.
