# Supplementary material for: Design of an Interpenetrating Polymeric Network Hydrogel Made of Calcium-Alginate from a Thermos-Sensitive Pluronic Template as a Thermal-Ionic Reversible Wound Dressing
Source: Polymers (Basel). 2020 Sep 18;12(9):2138. doi: 10.3390/polym12092138 (PMC7570184; doi:10.3390/polym12092138)
Supplement: Supplementary file 1 [file polymers-12-02138-s001.pdf]

polymers

Design of an interpenetrating polymeric network hydrogel made of calcium-alginate from a thermos-sensitive pluronic template as a thermal-ionic reversible wound dressing

Hsiao-Ying Chou[1], Chang-Chih Weng[1], Juin-Yih Lai[1,2,3], Shuian-Yin Lin[4]* and Hsieh-Chih Tsai[1,2,3,]*

[1]Graduate Institute of Applied Science and Technology, National Taiwan University of Science and Technology, Taipei, Taiwan
[2]Advanced Membrane Materials Center, National Taiwan University of Science and Technology, Taipei, Taiwan
[3]R&D Center for Membrane Technology, Chung Yuan Christian University, Chungli, Taoyuan, Taiwan
[4]Biomedical Technology and Device Research Center, Industrial Technology Research Institute, Hsinchu, Taiwan
* Correspondence: h.c.tsai@mail.ntust.edu.tw and Lilias@itri.org.tw; Tel.: +886-2-2730-3779; Fax: +886-2-2730-3733

Supporting information

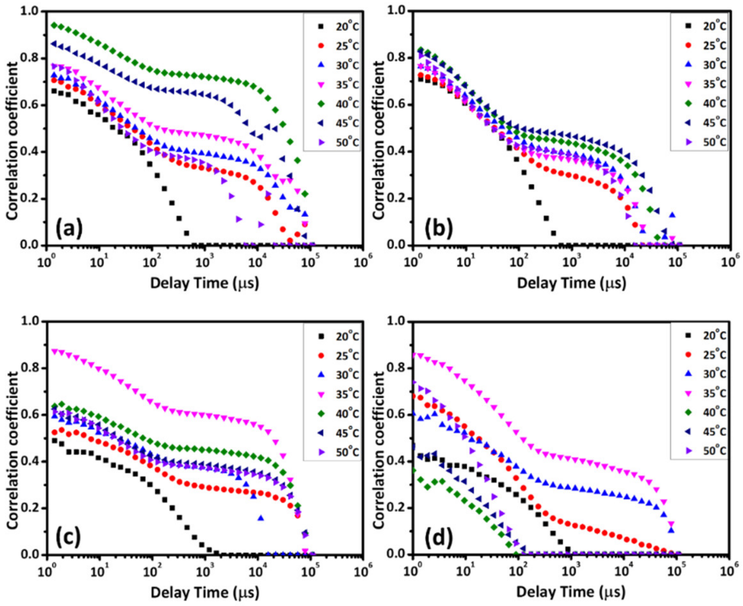

Figure 1. The effect of the sol-gel transition behavior at a different temperature from 20°C to 50°C investigate after diverse content sodium alginate blended into thermos-sensitive PF127. Each of composite hydrogel composed of PF127/SA 15/0.25, 15/0.5, 15/1, and 15/1.5 with various weights per volume concentration (% w/v). The correlation functions of DLS studies typically exhibits an exponential decay.

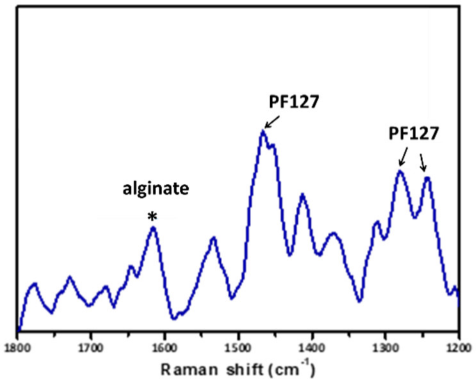

Figure 2. 2D Raman spectrum of PF127/SA IPN hydrogel, consist of ionic cross-linked and thermos network.

© 2020 by the authors. Licensee MDPI, Basel, Switzerland. This article is an open access article distributed under the terms and conditions of the Creative Commons Attribution (CC BY) license (http://creativecommons.org/licenses/by/4.0/).
